# Exogenous L-carnitine ameliorates burn-induced cellular and mitochondrial injury of hepatocytes by restoring CPT1 activity

**DOI:** 10.1186/s12986-021-00592-x

**Published:** 2021-06-24

**Authors:** Pengtao Li, Zhengguo Xia, Weichang Kong, Qiong Wang, Ziyue Zhao, Ashley Arnold, Qinglian Xu, Jiegou Xu

**Affiliations:** 1 Department of Burns, The First Affiliated Hospital of Anhui Medical University, No. 218 Jixi Road, Hefei, 230022 Anhui China; 2 Department of Wound Repair and Plastic and Aesthetic Surgery, The Fourth Affiliated Hospital of Anhui Medical University, No. 100 Huaihai Road, Xinzhan District, China; 3 Department of Immunology, School of Basic Medical Sciences of Anhui Medical University, No. 81 Meishan Road, Hefei, 230032 Anhui China; 4 International College of Anhui Medical University, No. 81 Meishan road, Hefei, 230032 Anhui China

**Keywords:** Carnitine, Burn, Mitochondrial injury, CPT1

## Abstract

**Background:**

Impaired hepatic fatty acid metabolism and persistent mitochondrial dysfunction are phenomena commonly associated with liver failure. Decreased serum levels of L-carnitine, a amino acid derivative involved in fatty-acid and energy metabolism, have been reported in severe burn patients. The current study aimed to evaluate the effects of L-carnitine supplementation on mitochondrial damage and other hepatocyte injuries following severe burns and the related mechanisms.

**Methods:**

Serum carnitine and other indicators of hepatocytic injury, including AST, ALT, LDH, TG, and OCT, were analyzed in severe burn patients and healthy controls. A burn model was established on the back skin of rats; thereafter, carnitine was administered, and serum levels of the above indicators were evaluated along with Oil Red O and TUNEL staining, transmission electron microscopy, and assessment of mitochondrial membrane potential and carnitine palmitoyltransferase 1 (CPT1) activity and expression levels in the liver. HepG2 cells pretreated with the CPT1 inhibitor etomoxir were treated with or without carnitine for 24 h. Next, the above indicators were examined, and apoptotic cells were analyzed via flow cytometry. High-throughput sequencing of rat liver tissues identified several differentially expressed genes (*Fabp4, Acacb, Acsm5,* and *Pnpla3*) were confirmed using RT-qPCR.

**Results:**

Substantially decreased serum levels of carnitine and increased levels of AST, ALT, LDH, and OCT were detected in severe burn patients and the burn model rats. Accumulation of TG, evident mitochondrial shrinkage, altered mitochondrial membrane potential, decreased ketogenesis, and reduced CPT1 activity were detected in the liver tissue of the burned rats. Carnitine administration recovered CPT1 activity and improved all indicators related to cellular and fatty acid metabolism and mitochondrial injury. Inhibition of CPT1 activity with etomoxir induced hepatocyte injuries similar to those in burn patients and burned rats; carnitine supplementation restored CPT1 activity and ameliorated these injuries. The expression levels of the differentially expressed genes *Fabp4, Acacb, Acsm5,* and *Pnpla3* in the liver tissue from burned rats and etomoxir-treated hepatocytes were also restored by treatment with exogenous carnitine.

**Conclusion:**

Exogenous carnitine exerts protective effects against severe burn-induced cellular, fatty-acid metabolism, and mitochondrial dysfunction of hepatocytes by restoring CPT1 activity.

**Supplementary Information:**

The online version contains supplementary material available at 10.1186/s12986-021-00592-x.

## Background

Severe burns, which are a type of extreme trauma, can affect nearly every organ and lead to substantial morbidity and mortality [1]. Many factors, such as ischemic hypoxic injury [2], uncontrolled inflammation [3], and the influence of bacterial toxins [4], among others, contribute to the impaired organ function observed after a burn. Severe burn-induced liver damage is a result of impaired fatty acid metabolism and persistent mitochondrial dysfunction [5]. If the injury progresses, hepatic steatosis or necrosis can occur, which is associated with a poor prognosis [6]. However, the molecular mechanisms underlying these changes are unclear.

Under normal circumstances, increased levels of free fatty acids stimulate β-oxidation. However, in patients with severe trauma, this response is inhibited. One of the mechanisms underlying this inhibition involves an increase in the glycolysis rate, which elevates the level of malonyl-CoA and can block the activity of carnitine palmitoyltransferase (CPT) 1 (CPT1), a key enzyme that is essential for the transport of long-chain fatty acids into mitochondria for β-oxidation [7, 8]. The pathogenesis of post-burn liver injury is thought to start with the abnormal accumulation of lipids in the liver due to stress conditions, such as metabolic disorders and imbalanced nutrient absorption. Excessive accumulation of lipids in the liver may lead to irreversible lipotoxicity and impaired mitochondrial function, and may in turn cause energy deficiency, endoplasmic reticulum stress, and the accumulation of reactive oxygen species (ROS) [9–11], which ultimately induce hepatocytic apoptosis.

Extreme trauma, such as severe burns, can lead to a decrease in plasma carnitine and an increase in carnitine excretion [12], accompanied by increased triglyceride (TG) levels, hyperlipidemia, and decreased levels of ketone bodies, particularly β-hydroxybutyrate (β-HB) [5]. Thus, the liver dysfunction that occurs in cases of severe trauma is likely associated with carnitine deficiency. Carnitine is a vitamin-like amino acid derivative widely present in organisms that plays an essential role in regulating fatty acid metabolism by functioning as a cofactor of CPT and is also associated with redox reactions and inflammation signaling [13]. Fatty acid catabolism in the mitochondria is an important pathway for obtaining energy and redox products, which are essential for the normal structure and function of the mitochondrion and the cell. Endogenous carnitine is synthesized in the liver and kidneys [14]. However, carnitine deficiency in severe trauma patients will likely exacerbate the impaired liver function without exogenous carnitine supplementation [14]. Despite the widely reported beneficial effects of carnitine as a food supplement, the underlying molecular mechanisms remain unclear.

In this study, the effects of exogenous carnitine supplementation on fatty acid metabolism, hepatocytic injury, and mitochondrial dysfunction were observed in severely burned rats. The results showed that exogenous carnitine supplementation exerted protective effects against hepatocytic injury and mitochondrial dysfunction by reversing the reduction in CPT1 activity. Inhibition of CPT1 activity in cultured hepatocytes induced similar hepatocytic injury and mitochondrial dysfunction, which could be restored by carnitine treatment. These results indicate that carnitine deficiency results in decreased CPT1 activity and subsequent mitochondrial dysfunction and hepatocytic injury. Therefore, our results provide a theoretical basis for exogenous carnitine supplementation as a strategy to reduce liver injury in patients with severe burns.

## Methods

### Patients with severe burns and healthy subjects

The study was conducted in accordance with the ethical principles of the Declaration of Helsinki and was approved by the local Ethics Committee of the First Affiliated Hospital of Anhui Medical University. Written informed consent was obtained from all study participants.

Twenty patients with severe burns who were hospitalized in the Department of Burn Surgery at the First Affiliated Hospital of Anhui Medical University (Hefei, China) from May 2017 to August 2018 were included in this study. The inclusion criteria werepatients with burns on more than 30% of their total body surface area (TBSA), aged 18–65 years old, admitted to the hospital within 6 h after the burn injury, and no evident disease in any major organ, including the liver. The exclusion criteria were evident shock and severely delayed resuscitation. Laboratory data and sera from healthy subjects (n = 10) were obtained from the Center of Healthy Examination of the First Affiliated Hospital of Anhui Medical University.

### Rat burn model and carnitine supplementation

Male Sprague‐Dawley rats weighing 210–260 g were housed in wire-bottom cages under a 12-h light/dark cycle and acclimated for 7 days before starting the experiments. All animals received food and water ad libitum for the entire study period. A well-established method [15] was used to induce 30% TBSA burns. All animals were injected with buprenorphine (0.05 mg/kg body weight, intramuscular) and pentobarbital sodium (40 mg/kg body weight, intraperitoneally) before the burn. After the burn, the rats were immediately resuscitated with lactated Ringers solution (40 ml/kg, intraperitoneally) and then euthanized at 12, 24, 48, and 72 h post burn.

Carnitine (300 mg/kg body weight) was injected immediately after burn injury via the caudal vein and daily thereafter for 2 days. At 24 h after the last treatment, the rats were euthanized as described above.

Blood samples were obtained from each rat for biochemical analysis, and slices of the liver were obtained and frozen at − 80 °C and/or fixed in 4% paraformaldehyde for later use. All animal procedures were approved by the Animal Care and Use Committee of Anhui Medical University.

### Biochemical analyses

The activity levels of aspartate aminotransferase (AST), alanine transaminase (ALT), lactate dehydrogenase (LDH), and TG levels in the serum and cell culture supernatants were measured using an auto-analyzer (Modular DPP; Roche, Switzerland).

Serum concentrations of carnitine, β-HB, and ornithine carbamoyltransferase (OCT) in the model rats and human burn patients were determined using Immunoassay Kits (Jiancheng Bioengineering Institute of Nanjing, Nanjing, China) according to the manufacturer’s instructions.

### Mitochondrial isolation

Liver tissue (300 mg) was cut up on ice and transferred (10% w/v) to separation buffer (0.33 mM sucrose, 0.025 mM EDTA, 15 mM Tris–HCl, pH 7.4). The tissues were gently homogenized with a homogenizer (2–3 times) and then centrifuged at 800 × *g* for 5 min. The supernatant was collected and centrifuged again at 8200 × *g* for 10 min to pellet the mitochondria.

### Apoptosis assay

To assess apoptosis of hepatocytes in the liver tissues, terminal deoxyuridine nick end labeling (TUNEL) staining was performed using a kit (Roche, Switzerland) according to the manufacturer’s instructions. Briefly, sections of the liver from six rats of each of the 24 h groups (control, burn, and burn + carnitine) were stained for apoptotic cells, and 10 randomly selected fields of each section were photographed to count the TUNEL-positive cells. The data were quantified as the percentage of apoptotic cells per hundred hepatocytes.

To assess apoptotic cells in vitro, the hepatocyte cell line HepG2 was cultured in a 6-well plate at a density of 4 × 10^5^ cells/well and incubated overnight at 37 °C. After 24 h of treatment (as indicated in the section below), the cells were collected and stained using the FITC Annexin V Apoptosis detection kit (Bestbio, Shanghai, China). Apoptotic HepG2 cells were analyzed via flow cytometry (FACS Calibur FCM; Becton–Dickinson, San Jose, CA, USA).

### Cell culture and treatments

HepG2 cells obtained from ATCC (Manassas, VA, USA) were cultured in DMEM (Biological Industries, Shanghai, China) containing 10% fetal bovine serum (FBS; Every Green Bio, Zhejiang, China) in 5% CO_2_ at 37 °C. When the cells reached 70–80% confluence, the medium was replaced with fresh medium containing the following additives: 10% FBS, 10% FBS + 100 μmol/L etomoxir (Selleck, USA), or 10% FBS + 100 μmol/L etomoxir + 200 μmol/L L-carnitine (MedChemExpress, China), and the cells were cultured for another 24 h. After treatment, the culture media were collected, and the cells were harvested for subsequent analyses.

### Examination of mitochondrial structure and hepatocyte function

The morphology and structure of the mitochondria in the liver tissues were examined via transmission electron microscopy (TEM; JEM1400). Briefly, liver tissues (2 × 2 × 2 mm^3^) were excised and fixed with 1% osmic acid for 2 h. The tissues were then dehydrated in a graded series of alcohol followed by propylene oxide and embedded in epoxy resin. Sections were cut (70 nm thick) using an ultra-microtome, collected on uncoated grids, stained with uranyl acetate and lead citrate, and then processed for TEM observation.

The mitochondrial membrane potential (∆Ψm) in HepG2 cells and liver tissues were examined using a mitochondrial membrane assessment kit (Bestbio, Shanghai, China) according to the manufacturer’s instructions. HepG2 cells were cultured overnight in a 6-well plate at a density of 3 × 10^5^ cells/well. After 24 h of treatment with etomoxir or etomoxir plus carnitine, the adherent cells and cultured cells were co-incubated with JC-1 and detected via flow cytometry. FlowJo software was used for data analysis.

The ATP content of HepG2 cells was determined using the Enhanced ATP Assay Kit (Beyotime, China) according to the manufacturer’s instructions, and the results are expressed as a percentage of the control.

### Determination of oxygen consumption rate (OCR)

The OCR in HepG2 cells and liver tissues was determined using the OCR assessment kit (Bestbio, Shanghai, China) according to the manufacturer’s protocol. Briefly, the cells and mitochondria treated as described above were plated in a 96-well plate, and 150 μl of fresh medium was added. Thereafter, 10 μl of the oxygen fluorescent probe was added, followed by blocking with 100 μl of oxygen blocking solution. Next, the OCR was calculated based on the optical density at 468 nm.

### Detection of intracellular triglyceride levels

HepG2 cells were cultured for 24 h in 6-well plates and then exposed to the indicated treatments. Cellular lipid accumulation was visualized via Oil Red O staining.

Intracellular TG content was measured using a triglyceride assay kit (Nanjing Jiancheng Bioengineering Institute, Nanjing, Jiangsu, China) according to the manufacturer’s instructions.

### CPT1 activity assay

Mitochondrial membrane proteins were extracted using a mitochondrial protein extraction kit (Bestbio, Shanghai, China). The protein concentrations in the supernatants were determined using the BCA protein assay (Bestbio, Shanghai, China) according to the manufacturer’s instructions. To analyze CPT1 activity, a reaction mixture containing Tris-buffer (100 mM, pH 8.0, 0.1% Triton X-100, 1 mM EDTA), 0.01 mM palmitoyl CoA, and 0.5 mM DTNB was added to each sample, and then the absorbance at 405 nm was measured using a microplate reader. Thereafter, L-carnitine (1.25 mM) was added, incubated for 30 min, and the absorbance at 405 nm was measured.

### RNA isolation and gene expression analysis

Total RNA was extracted from the collected liver tissues and cells using TRIzol reagent (Magen) and was reverse-transcribed using 5 × HiscriptII qRT SuperMix for qPCR (Vazyme, Nanjing, China) according to the manufacturers’ instructions. PCR was conducted using the following cycling parameters: 95 °C for 5 min, followed by 40 cycles of 95 °C for 10 s and 60 °C for 30 s. Quantitative real-time PCR was performed for the target genes carnitine palmitoyltransferase 1α (*Cpt1α*), fatty acid binding protein 4 (*Fabp4)*, acyl-CoA synthetase medium chain family member 5 (*Acsm5)*, patatin-like phospholipase domain-containing protein 3 (*Pnpla3)*, and acetyl-coenzyme A carboxylase (*Acacb)* and was normalized to the levels of *GAPDH* mRNA in the same sample. The average of three independent analyses for each gene and sample was calculated using the comparative cycle threshold (∆∆Ct) method. The sequences of the primers used are presented in an additional file (Additional file 1: Table S1).

### Western blot analysis

Cells and liver tissues were lysed with RIPA buffer containing 50 mM Tris, pH 7.4, 150 mM NaCl, 1% sodium deoxycholate, 1% Triton X-100, 0.1% SDS, and 1 × protease inhibitor cocktail. The lysates were then centrifuged at 14,000 × *g* for 20 min at 4 °C. The protein concentrations of the supernatants were assessed using the BCA assay kit (Beyotime, China) according to the manufacturer’s protocol. Lysates containing equal quantities of protein were resolved by 10% SDS-PAGE and then transferred to a PVDF membrane (Millipore, USA) in transfer buffer for 90 min at 200 mA. Subsequently, the membranes were blocked with 5% nonfat milk in TBST (1.5 mM Tris-Base, 8.5 mM Tris–HCl, pH 7.4, 150 mM NaCl, and 0.1% Tween 20) for 2 h, and then incubated at 4 °C overnight with the following primary antibodies: anti-CPT1 (Abcam, USA), anti-Bax, and anti-Bcl-2 (both from Proteintech, Wuhan, China). An antibody against GAPDH (Absin, China) was used as an internal control. The membranes were then washed four times with TBST for 20 min each and then incubated with anti-rabbit or anti-mouse secondary antibodies (1:10,000 in TBST; ZSGB-BIO, Beijing, China) for 1.5 h at room temperature. The immunoreactive protein bands were visualized using the ECL kit (Thermo Fisher Scientific, USA).

### Statistical analysis

Results are expressed as the mean ± standard deviation (SD) and were analyzed using SPSS 21.0 statistical software (SPSS Inc., Chicago, IL, USA). Statistical analysis of multiple groups was performed using one-way analysis of variance followed by Bonferroni post hoc tests or Tamhane T2 test. Student’s *t*-test was used to compare the differences between two groups. A two-tailed *P* value less than 0.05 was considered to be statistically significant.

## Results

### Changes in carnitine levels, indicators of hepatocyte and mitochondrial injury, and fatty acid metabolism in severe burn patients

Sera from 20 patients with early stage severe burns and 10 healthy controls were collected, and parameters reflecting hepatocellular and mitochondrial injury, fat metabolism, and carnitine levels were evaluated. The clinicopathological features of the burn patients are summarized in an additional file (Additional file 1: Table S2). As shown in Table 1, carnitine serum levels were significantly lower in severe burn patients than in healthy controls. Compared with the healthy controls, the levels of various indicators of hepatocyte injury, including ALT, AST, and LDH, were significantly increased in severe burn patients. An increase in serum TG, decreases in serum β-HT and free carnitine, and increased OCT release all indicated abnormalities in fatty acid catabolism and mitochondrial injury in severe burn patients. These results suggest that carnitine deficiency may be a factor associated with the liver injury observed in patients with severe burns.

**Table 1:** Serum parameters in healthy subjects and burn patients

Parameters	Healthy subjects (n = 10)	Burn patients (n = 20)	*p *value
ALT (U/L)	15.70 ± 5.56	34.05 ± 7.44	< 0.0001
AST (U/L)	17.20 ± 6.03	58.70 ± 13.65	< 0.0001
LDH (U/L)	158.60 ± 18.21	530.10 ± 199.10	< 0.0001
TG (mmol/mL)	0.99 ± 0.27	1.15 ± 0.29	n.s
OCT (ng/mL)	7.47 ± 0.73	113.48 ± 34.11	< 0.0001
b-HB (mmol/L)	108.80 ± 10.54	14.27 ± 5.48	< 0.0001
Carnitine ug/L)	48.10 ± 7.13	11.08 ± 4.45	< 0.0001

Serum ALT, AST, LDH, TG, OCT, β-HB, and carnitine levels were analyzed using an auto-analyzer after burn injury (> 30% TBSA, within 24 h). Data are expressed as means ± SD. ns, not significant, Student’s t-test was used to determine the significance of differences (*P-*value)

### Effects of carnitine supplementation on hepatocyte injury in burned rats

**Fig. 1:**
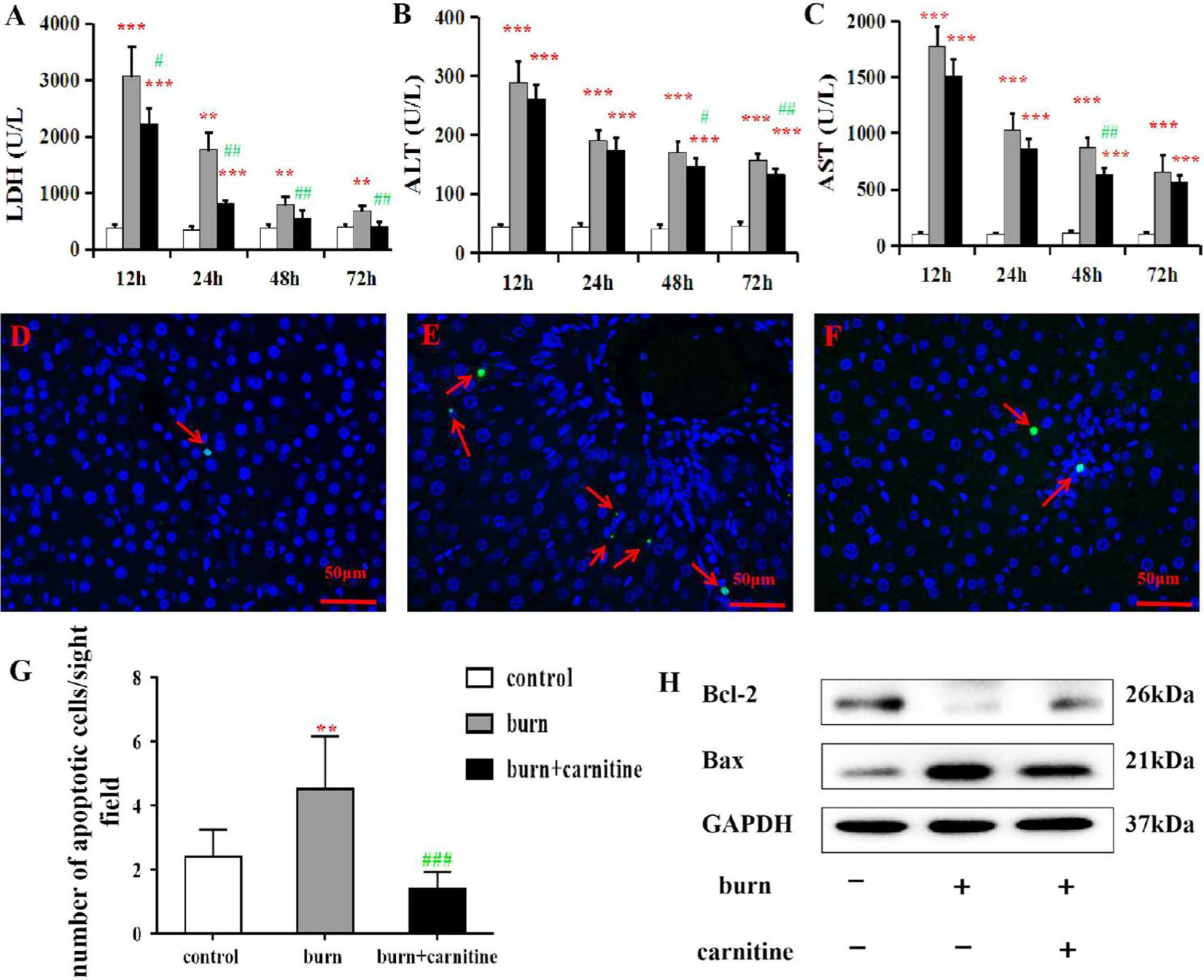
Effects of carnitine supplementation on hepatocyte injury in burned rats. Serum levels of LDH (**A**), ALT (**B**), and AST (**C**) were detected in the control, burn, and burn + carnitine groups of rats. Representative images of TUNEL-stained liver tissues from the control (**D**), burn (**E**), and burn + carnitine (**F**) rats are shown. The numbers of TUNEL-positive cells in 10 randomly selected fields are shown in (**G**). (**H**) The levels of Bax and Bcl-2 proteins were measured via western blotting. Data are presented as means ± SD. **, *P* < 0.01 and ***, *P* < 0.001, versus the control group; #, *P* < 0.05; ##, *P* < 0.01, and ###, *P* < 0.001 versus the burn group

To investigate whether carnitine supplementation can protect effects on liver function, we first established a rat burn model according to a well-recognized method [15]. Although no pathological changes were observed in the liver tissue (Additional file 1: Fig. S1), the biochemical parameters in the burned rats were remarkably similar to those observed human burn patients. Serum carnitine was significantly decreased at 12 h post burn and then gradually increased (Additional file 1: Fig. S2). As shown in Fig. 1A–C, the activity levels of ALT, AST, and LDH were increased at 12, 24, 48, and 72 h post burn, with the highest activities at 12 h. There were an increased number of apoptotic hepatocytes in the liver tissues of the burned rats, as revealed by TUNEL staining (Fig. 1D–G) and western blotting (Fig. 1H). Carnitine supplementation reduced the activity levels of ALT, AST, and LDH, as well as hepatocyte apoptosis (Fig. 1A, B, C, G, and H).

Similarly, dysfunction of fatty metabolism was also observed in the burn rats, as shown by the increase in TG accumulation in hepatocytes, as measured via Oil Red O staining (Additional file 1: Fig. S3 A, B, and D) and the TG levels measured in hepatic tissue (Additional file 1: Fig. S3 E). Treatment with exogenous carnitine decreased all these parameters in the liver tissue (Additional file 1: Fig. S3 C, D, and E). These results indicated that exogenous carnitine had a protective effect against hepatocytic injury and fatty metabolism dysfunction.

### Effects of carnitine supplementation on mitochondrial injury in the hepatocytes of burned rats

**Fig. 2:**
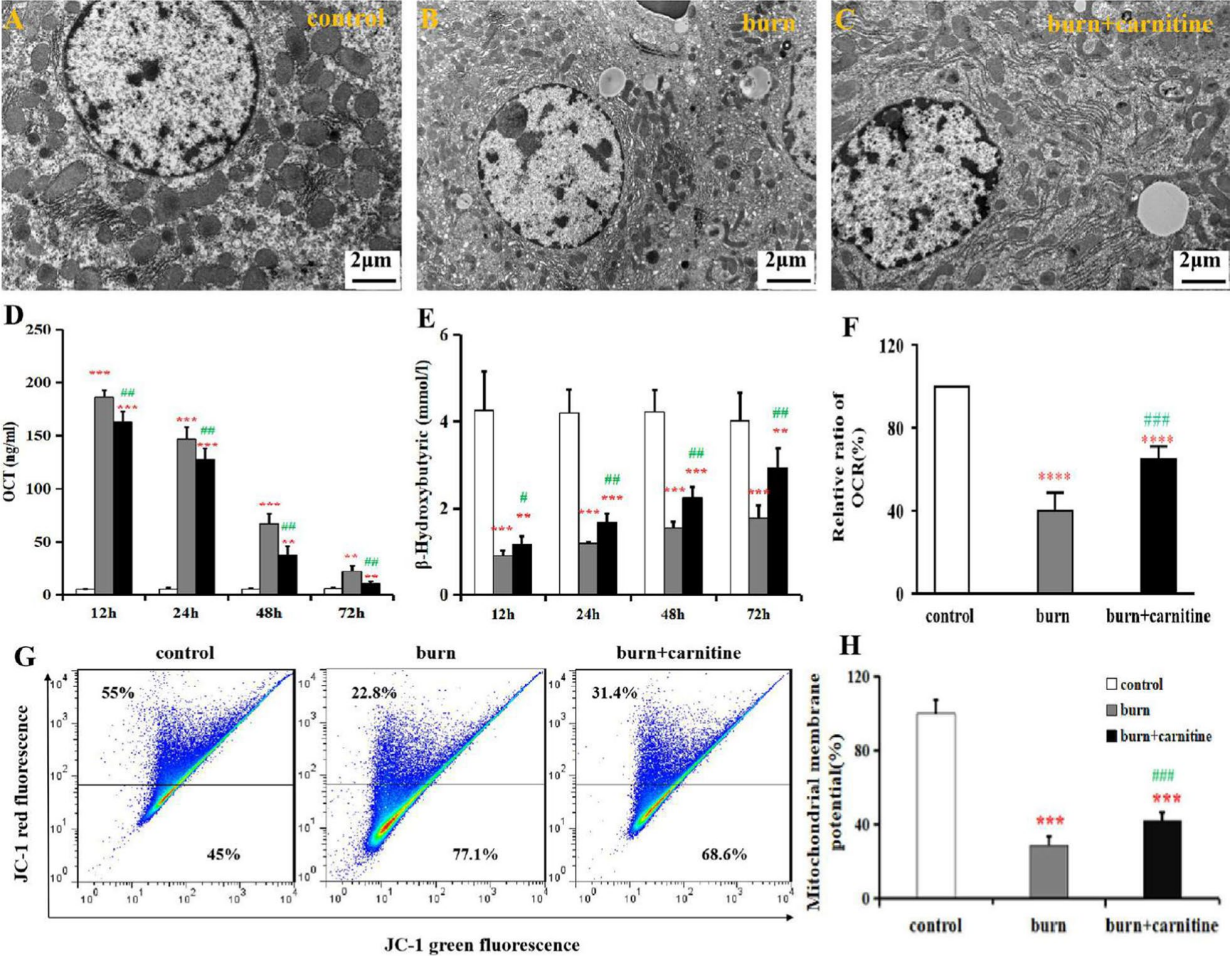
Effects of carnitine supplementation on mitochondrial injury in the hepatocytes of burned rats. Representative transmission electron microscopy images of hepatocytes from normal (**A**), burned rats (**B**), and carnitine-treated burned rats (**C**) are shown. Serum levels of OCT (**D**) and β-HB (E) are shown. The oxygen consumption rate (OCR) of the mitochondria in the liver tissues is shown in (**F**). Changes in the mitochondrial membrane potential (∆Ψm) in the liver tissues of the normal, burn, and burn + carnitine rats were analyzed via flow cytometry, and the results are shown in (**G**) and (**H**). The relative mitochondrial membrane potential in the control is set at 100%. Data are presented as means ± SD. *, *P* < 0.05; **,* P* < 0.01; and ***,* P* < 0.001 versus the control group; ##, *P* < 0.01 versus the burn group

Observations made using TEM showed that the mitochondria in the hepatocytes of burned rats had shrunk (Fig. 2A, B). Simultaneously, increased levels of OCT, a key enzyme in hepatocytic mitochondria that is associated with urea synthesis, were released into the serum of burned rats (Fig. 2D). These results are indicative of burn-induced structural injury of the mitochondria. Functional changes in the hepatocytic mitochondria of the burned rats included decreases in β-HB generation (Fig. 2E), the OCR (Fig. 2F), and mitochondrial membrane potential (Fig. 2G, H). In contrast, ATP production was not changed (data not shown). Carnitine supplementation significantly ameliorated these indicators of structural and functional injury to the hepatocytic mitochondria (Fig. 2C–G).

**Fig. 3:**
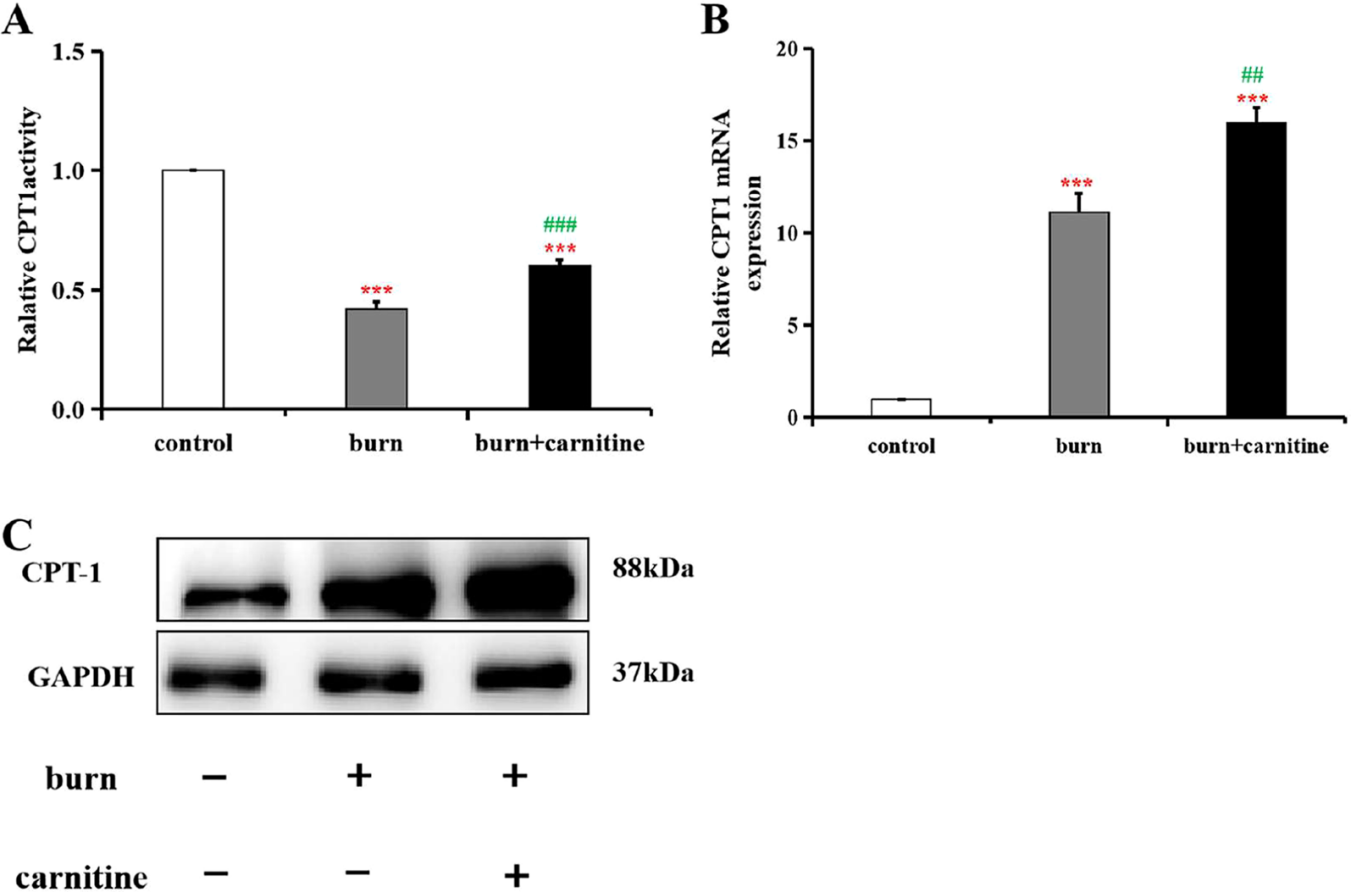
Changes in CPT1 activity and CPT1α mRNA expression levels in rat liver tissues. Relative mitochondrial CPT1 activity levels in the liver tissues of the rats were determined at 24 h post burn, and the control group was assigned a value of 1 (**A**) Relative mRNA and protein expression levels of CPT1 a-chain in the rat liver tissues was assessed via RT-PCR (**B**) and western blotting (**C**), respectively. Data are presented as means ± SD, n = 6 rats. *,* P* < 0.05; **,* P* < 0.01; and ***,* P* < 0.001 versus the control group; #,* P* < 0.05; *##*,* P* < 0.01; and *###*,* P* < 0.001 versus the burn group

Carnitine is a cofactor of CPT1, a key enzyme that is essential for the transport of long-chain fatty acids into mitochondria for β-oxidation and is located on the membrane of hepatocytic mitochondria. The observed carnitine deficiency, liver injury in both human burn patients and burn model rats, and the protective effects of exogenous carnitine caused changes in CPT activity and/or expression. Measurement of CPT1 activity revealed that it was indeed lower in the burned rats than in the controls and that this was partially reversed by treatment with exogenous carnitine (Fig. 3A), indicating that the burn-induced liver injury was due, at least in part, to carnitine deficiency and reduced CPT1 activity. Interestingly, quantitative RT-PCR (Fig. 3B) and western blotting (Fig. 3C) showed that CPT mRNA and protein levels were increased in the livers of the burned rats. This was probably due to compensative expression.

### Hepatocytic injury induced by CPT1 inhibition and the protective effects of carnitine in vitro

**Fig. 4:**
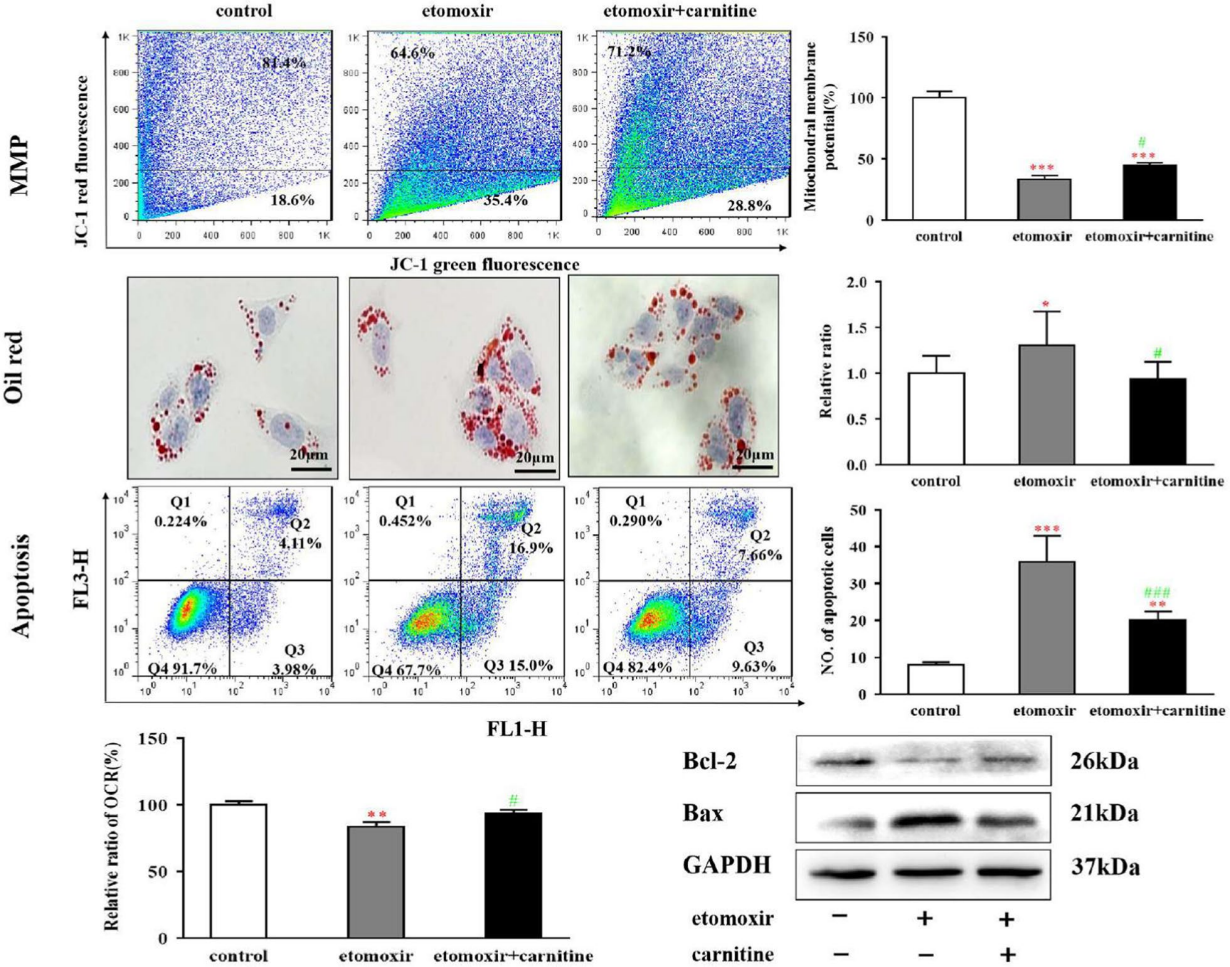
Effects of carnitine supplementation on mitochondrial membrane potential, lipid accumulation, and apoptosis of HepG2 cells. HepG2 cells were treated with or without 0.1 mM etomoxir or with 0.1 mM etomoxir + 0.2 mM L-carnitine for 24 h. Mitochondrial membrane potential (MMP, upper left panel) was analyzed using JC-1 staining and flow cytometry, and relative MMP is shown in the upper right panel. TG accumulation was evaluated via Oil Red O staining (middle of the left panel), and the relative density is shown in the middle of the right panel. Apoptotic HepG2 cells were stained with PI and annexin V and then analyzed via flow cytometry (lower portion of the left panel), and the number of apoptotic cells is shown in the lower part of the right panel. The oxygen consumption rate (OCR) was analyzed using a multifunctional microplate reader. Protein levels of Bax and Bcl-2 in HepG2 cells were analyzed after 24 h of treatment. Data from three independent experiments are shown as means ± SD. *,* P* < 0.05 and ***,* P* < 0.001 versus the control group; #,* P* < 0.05 and *###*,* P* < 0.001 versus the etomoxir group

To confirm whether inhibition of CPT1 caused the cellular and mitochondrial injuries observed in the burn model rats and burn patients, etomoxir, a specific CPT1 inhibitor, was added to the culture medium of the hepatocyte cell line HepG2. Etomoxir inhibited CPT1 activity to 75% of the levels in the untreated control cells (Additional file 1: Fig. S4A), and CPT1 α-chain mRNA expression levels were compensatively increased (Additional file 1: Fig. S4B). Treatment with etomoxir also led to increases in the activities of AST, ALT, and LDH in the culture medium (Table 2) as well as apoptosis (Fig. 4), indicating that cellular injury was induced by etomoxir. In addition, increased levels of OCT were released into the culture medium following the addition of etomoxir (Table 2). Measurement of the mitochondrial membrane potential and OCR showed significant decreases in etomoxir-treated HepG2 cells (Fig. 4). These results demonstrated that structural and functional injuries were induced by etomoxir treatment. Evaluation of TG levels in HepG2 homogenates using a biochemical assay and in the cytoplasm via Oil Red O staining revealed that etomoxir treatment resulted in TG accumulation (Table 2 and Fig. 4). In brief, the cellular and mitochondrial injuries in HepG2 induced by etomoxir were remarkably similar to those observed in the burn model rats and burn patients.

**Table 2:** Indicators of cell injury and metabolism in hepatocytes

Parameters	Control	Etomoxir	Etomoxir + carnitine	*p *values
ALT (U/L)	3.35 ± 0.98	8.16 ± 0.90	5.06 ± 1.36	*P*^a^ < 0.01, *P*^b^ < 0.05
AST (U/L)	5.23 ± 0.54	10.50 ± 1.28	7.15 ± 1.36	*P*^a^ < 0.01, *P*^b^ < 0.05
LDH (U/L)	16.36 ± 2.78	33.53 ± 3.02	20.21 ± 5.82	*P*^a^ < 0.01, *P*^b^ < 0.05
TG (mmol/mL)	0.25 ± 0.02	0.51 ± 0.04	0.21 ± 0.01	*P*^a^ < 0.0001, *P*^b^ < 0.0001
OCT (ng/mL)	49.40 ± 8.73	109.06 ± 10.32	78.82 ± 8.59	*P*^a^ < 0.01, *P*^b^ < 0.05
b-HB (mmol/L)	93.22 ± 5.84	43.62 ± 9.71	73.40 ± 8.54	*P*^a^ < 0.01, *P*^b^ < 0.01

HepG2 cells were treated with or without 0.1 mM etomoxir or with 0.1 mM etomoxir + 0.2 mM carnitine for 24 h. After treatment, ALT, AST, LDH, TG, OCT, and β-HB levels in the culture medium were evaluated using an auto-analyzer. Student’s t-test was used to calculate the *P* values

^a^Etomoxir versus control

^b^Etomoxir + carnitine versus etomoxir

The addition of carnitine to the culture medium of the etomoxir-treated HepG2 cells partially reversed the reduced CPT1 activity levels and further increased the mRNA and protein levels of CPT1 α-chain (Additional file 1: Fig. S4A, B, C). Treatment with carnitine also improved the indicators of the cellular and mitochondrial injuries induced by etomoxir (Table 2 and Fig. 4), although these parameters did not reach the levels of the control.

### Gene expression analysis via high-throughput sequencing in burned rats

To further clarify the effect of carnitine supplementation on gene expression in liver tissues, high-throughput sequencing of transcribed mRNAs was conducted. A total of 22 differentially expressed genes were screened in the normal control, burn, and burn + carnitine groups (Additional file 1: Fig. S5A). Among them were four genes are related to lipid metabolism: *Acacb*, *Acsm5*, *Fabp4*, and *Pnpla3*. Confirmation of the expression levels of the four genes via quantitative PCR and PCR (Additional file 1: Fig. S5B, C) indicated that the expression levels of *Fabp4* were significantly increased, whereas the expression levels of *Acacb* and *Pnpla3* were decreased in the burn group. Treatment with carnitine decreased *Fabp4* expression. Interestingly, similar results were also obtained in etomoxir-treated and etomoxir + carnitine-treated HepG2 cells, (Additional file 1: Fig. S5D). Although the exact roles of these genes in burn-induced hepatocyte injury are unknown at present, exogenous carnitine was able to reverse the expression changes in these genes.

## Discussion

Carnitine deficiency is a common phenomenon in traumatic diseases and injuries such as severe burns and is manifested as a decrease in plasma carnitine and an increase in carnitine excretion [12, 16]. Enhanced renal clearance of carnitine [17] together with elevated consumption of free carnitine due to increased β-oxidation of fatty acids as an energy source [13] results in decreased carnitine storage in the body. Carnitine is a cofactor of CPT1 that is necessary for the transport of long-chain acyl-CoA from the cytosol to the mitochondria for β-oxidation. Therefore, carnitine deficiency leads to reduced CPT1 activity, which further affects fatty acid oxidation and subsequent ketone body generation. β-HB, which is one of the major ketone bodies, is a vital energy source for the brain, heart, skeletal muscles, kidneys, and other organs. We found that β-HB levels were reduced in burn patients and burned rats. Following severe trauma, CPT1 activity can also be inhibited by elevated malonyl CoA levels, possibly due to enhanced glycolysis [18, 19].

In the present study, we observed the protective effects of exogenous carnitine on liver injury in a rat model of severe burn. As expected, serum carnitine levels declined in burned rats. In addition, serum activity levels of LDH, ALT, and AST, which are indicators of hepatocyte injury, were abnormally increased in the burned rats and were remarkably similar to the levels detected insevere burn patients enrolled in this study. Supplementation with carnitine alleviated the cellular injury of hepatocytes. Similarly, considerable accumulation of TG in the liver tissue was detected in the rats at 24 h post burn, although there was no significant change in serum TG levels. These results are consistent with previous reports [5, 19]. Exogenous carnitine supplementation reduced the TG contents in liver tissue. The accumulation of TG in liver tissue is likely related to the lack of carnitine, which limits the transport and oxidation of long-chain fatty acids, and free fatty acid re-esterification to TG in hepatocytes, which is blocked from returning to the plasma in the form of very-low-density lipoprotein (VLDL). Simultaneously, excessive lipolysis in adipose tissue leads to increased levels of plasma fatty acids and VLDL-TG, which aggravates liver TG production and accumulation [19].

OCT is a specific enzyme present in hepatocyte mitochondria [20, 21]. After burns or other trauma, the presence of liver damage results in increased permeability of the membranes of liver cells and mitochondria. OCT can be released from the mitochondria into the blood, causing an increase in the serum OCT concentration. Therefore, OCT is a sensitive and specific indicator of mitochondrial damage in liver cells [22]. Consistent with these results, serum OCT levels were persistently increased in our burn model rats. The increase in OCT, together with the evident shrinkage of the mitochondria, as observed via TEM, reflected structural injury after burn. β-HB, which is a major ketone body, is a byproduct of fatty acid β-oxidation in the mitochondria. Decreased serum β-HB levels and altered mitochondrial membrane potential in the burned rats indicate functional injury of the mitochondria. Our results demonstrated that carnitine supplementation was able to ameliorate the structural and functional damage induced by burn. It is plausible that carnitine supplementation could restore the ketogenic ability of the liver and the stability of liver metabolism, thereby maintaining the structural and functional integrity of the hepatocyte mitochondria [23, 24].

Carnitine mainly functions as a cofactor of CPT1, and it increases the activity of CPT1 and β-oxidation [25]. This study showed that the mitochondrial CPT1 activity of rat liver cells was significantly decreased after severe burn. Supplementation with carnitine restored CPT1 enzyme activity, fatty acid metabolism, and mitochondrial function. Based on clinical observations and animal experiments, we hypothesized that the lack of carnitine in the body post burn combined with other factors would lead to a reduction in hepatocyte mitochondrial CPT1 activity, and reduced CPT1 activity would result in subsequent abnormal fatty acid metabolism and reduced ketogenic capacity in the mitochondria, thus leading to impairment of mitochondrial structural and functional integrity and hepatocyte viability. We mimicked this reduction in CPT1 activity in HepG2 cells by treating the cells with etomoxir, a specific inhibitor of CPT1 and fatty acid oxidation [26–28], to determine whether inhibition of CPT1 induces similar hepatocyte injuries to those observed in burn patients and the burn model rats. As expected, etomoxir-induced inhibition of CPT1 caused similar cellular, mitochondrial, and fatty acid metabolic dysfunctions, indicating that inhibition of CPT1 is a key factor in severe burn-induced hepatocyte damage. Interestingly, carnitine was able to reverse the effects of etomoxir-induced CPT1 inhibition and alleviate hepatocyte injuries. We also performed transcription high-throughput sequencing of liver tissues to compare gene the expression profiles of the control, burn, and burn + carnitine rats. Four differentially expressed genes associated with lipid metabolism, *Acacb*, *Acsm5*, *Fabp4*, and *Pnpla3*, were found. Quantitative RT-PCR confirmed the expression patterns of these genes in both the burned rats and etomoxir-treated HepG2 cells. Acetyl-CoA carboxylase beta, encoded by *Acacb*, is a mitochondrial enzyme that catalyzes the carboxylation of acetyl-CoA to generate malonyl-CoA, and through the production of malonyl-CoA, which allosterically inhibits CPT1 on the mitochondria and negatively regulates fatty acid oxidation. Acacb plays a central role in fatty acid metabolism and is associated with biotin deficiency, fatty liver disease, and type 2 diabetes mellitus [29]. Acsm5 catalyzes the activation of fatty acids by CoA to produce acyl-CoA in the first step of fatty acid metabolism [30]. Fabp4 binds to both long chain fatty acids and retinoic acid and delivers them to their cognate receptors in the nucleus. Its upregulation is related to non-alcoholic steatohepatitis [31] and other human diseases, such as cancer and metabolic disorders [32]. Pnpla3 specifically catalyzes coenzyme A-dependent acylation of 1-acyl-sn-glycerol 3-phosphate (2-lysophosphatidic acid/LPA) to generate phosphatidic acid, an important metabolic intermediate and precursor of both TGs and glycerophospholipids, and is associated with fatty liver disease [33]. Downregulated expression of Acacb, Acsm5, and Pnpla3 and upregulated expression of Fabp4 in the burned rats suggest that these genes are involved in burn-induced liver abnormalities, although the exact mechanisms need to be investigated. Interestingly, in our preliminary studies, we observed that introduction of Fabp4 into hepatocytes causes cell apoptosis.

Many studies have shown that early enteral and parenteral nutrition can reduce the incidence of infection and organ failure. Therefore, international guidelines call for the provision of early nutritional support [4, 5]. However, carnitine is not included in these guidelines. Our results indicate that early supplementation with carnitine would be helpful for the amelioration of liver injury in patients with severe burns or other severe trauma.

## Conclusions

Exogenous carnitine supplementation exerts protective and therapeutic effects against severe burn-induced dysfunction of fatty acid metabolism and cellular and mitochondrial injury of hepatocytes by restoring CPT1 activity and promoting CPT1 expression.

## Supplementary Information

**Additional file 1**. **Table S1.** List of primers used for RT-qPCR. **Tables S2.** Clinicopathological charecteristics of severe burn patients. **Fig. S1.** Representative H&E stained images of the liver tissues from rats with severeburns. **Fig. S2.** Serum carnitine levels in burned rats. **Fig. S3.** Effects of exogenous carnitine on hepatic TG levels in burned rats. **Fig. S4.** Effects of carnitine on CPT1 activity and CPT1 expression in vitro. **Fig. S5.** Gene expression analysis in burned rats using high-throughput sequencing.

## Data Availability

All data generated or analyzed during this research are included in this published article or are obtained from the corresponding author on reasonable request.

## Abbreviations

CPT1: Carnitine palmitoyltransferase 1
ROS: Reactive oxygen species
TG: Triglyceride
β-HB: β-Hydroxybutyrate
TBSA: Total body surface area
AST: Aspartate aminotransferase
ALT: Alanine transaminase
LDH: Lactate dehydrogenase
OCT: Ornithine carbamoyltransferase
TUNEL: Terminal deoxyuridine nick end labeling
FBS: Fetal bovine serum
TEM: Transmission electron microscopy
OCR: Oxygen consumption rate
Ct: Cycle threshold
Cpt1α: Carnitine palmitoyltransferase 1α
SD: Standard deviation
VLDL: Very-low-density lipoprotein
Acsm5: Acyl-CoA synthetase medium chain family member 5
Fabp4: Fatty acid binding protein 4
Pnpla3: Patatin-like phospholipase domain-containing protein 3
