# Supplementary material for: Exogenous L-carnitine ameliorates burn-induced cellular and mitochondrial injury of hepatocytes by restoring CPT1 activity
Source: Nutr Metab (Lond). 2021 Jun 24;18:65. doi: 10.1186/s12986-021-00592-x (PMC8223334; doi:10.1186/s12986-021-00592-x)
Supplement: Supplementary file 1 — Additional file 1. Table S1. List of primers used for RT-qPCR. Tables S2. Clinicopathological charecteristics of severe burn patients. Fig. S1. Representative H&E stained images of the liver tissues from rats with severeburns. Fig. S2. Serum carnitine levels in burned rats. Fig. S3. Effects of exogenous carnitine on hepatic TG levels in burned rats. Fig. S4. Effects of carnitine on CPT1 activity and CPT1 expression in vitro. Fig. S5. Gene expression analysis in burned rats using high-throughput sequencing. [file 12986_2021_592_MOESM1_ESM.pdf]

Supplementary Materials

Exogenous L-carnitine ameliorates burn-induced cellular and mitochondrial injury of hepatocytes by restoring CPT1 activity

Pengtao Li, Zhengguo Xia, Weichang Kong, Qiong Wang, Ziyue Zhao, Ashley Arnold, Qinglian Xu, and Jiegou Xu

*Corresponding authors. Email: xuqinglian@sina.com, and xujiegou@ahmu.edu.cn

This PDF file includes:

Table S1. List of primers used for RT-qPCR.

Tables S2.Clinicopathological charecteristics of severe burn patients.

Fig. S1. Representative H&E stained images of the liver tissues from rats with severe burns.

Fig. S2. Serum carnitine levels in burned rats.

Fig. S3. Effects of exogenous carnitine on hepatic TG levels in burned rats.

Fig. S4. Effects of carnitine on CPT1 activity and CPT1 expression in vitro

Fig. S5. Gene expression analysis in burned rats using high-throughput sequencing.

Table S1. List of primers used for RT-qPCR.

organism	name	Forward Primer sequence (5'-3')	Reverse primer sequence (5'-3')	Annealing temp.(°C)
Rat	Fabp4	tgaaaggcgtgacttctacaa	accaccaaatcccatcaagt	60
	Acacb	cctccaccattgtagcccag	tccctcctcca ccttca gag	60
	Acsm5	gcagcctgga ctctcttctc	ccacttgtccctcacgtcag	60
	Pnpla3	cttgaggaggcgagtctagc	cagcatggggaagtgggatc	60
	Cptl	tatcacctttgggagcgtggag	gagtcgaccgactagggagg	60
Human	Fabp4	cgtcatgaaaggcgtcactt	tgcaaatttcccatttccttcac	58
	Acacb	aggtcctgggaagagaggtc	gtagggagctctggatggga	60
	Acsm5	gaaggacctcaagtaccggc	gtgctctgtagaagcctccc	60
	Pnpla3	cttgaggaggcgagtctagc	cagcatggggaagtgggatc	60
	Cptl	gatcctggacaatacctcggag	ctccacagcatcaagagactgc	60

Tables S2. Clinicopathological characteristics of severe burn patients.

Patient	Gender	Age (years)	Hospitalization time from burns (hours)	Initial wound size
1	Male	55	5	80
2	Male	60	4	70
3	Female	44	4	70
4	Female	59	4	75
5	Female	35	5	40
6	Male	59	5	65
7	Male	41	5	50
8	Male	28	3	85
9	Female	62	2	47
10	Female	36	3	94
11	Male	*42*	5	94
12	Male	*50*	4	70
13	Female	*57*	3	80
14	Female	28	1	75
15	Male	52	*5*	70
16	Female	44	*2*	95
17	Female	41	5	85
18	Male	47	2	50
19	Female	41	3	65
20	Female	19	1	80

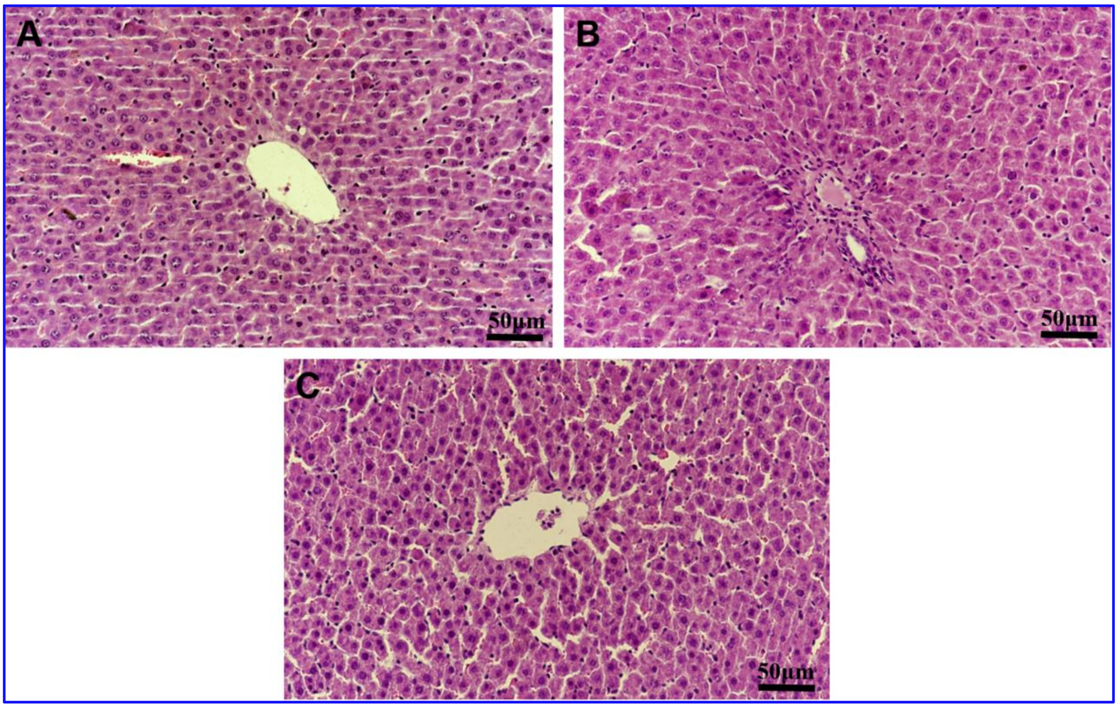

Fig. S1. Representative H&E stained images of the liver tissues from rats with severe burns

The rats were treated with or without 300 mg of carnitine immediately post burn, and the liver tissues were processed for histological examination. H&E-stained images of liver tissues from the control (A), burn (B), and burn+carnitine (C) group rats at 24 h post burn.

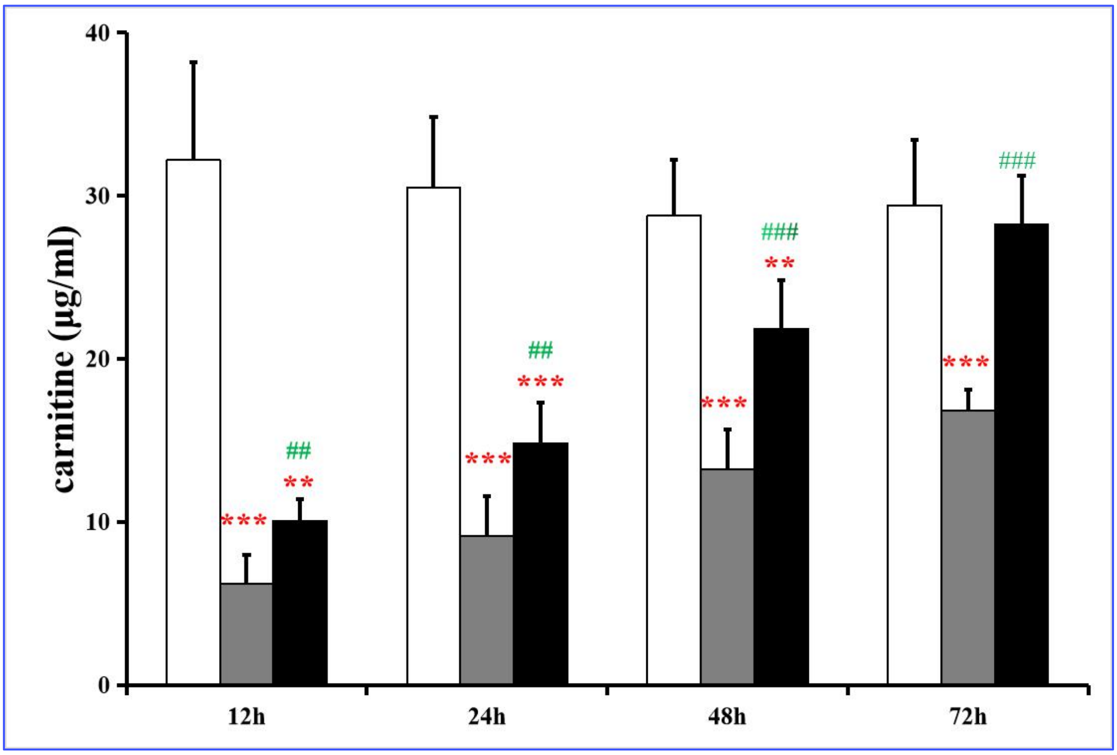

Fig. S2. Serum carnitine levels in burned rats

Serum carnitine levels were detected at 12, 24, 48, and 72 h post burn injury. Data are shown as means ± SD; n = 6 rats. **, $P < 0.01$ and ***, $P < 0.001$ vs. the control group; #, $P < 0.05$; ##, $P < 0.01$; and ###, $P < 0.001$ vs. the burn group.

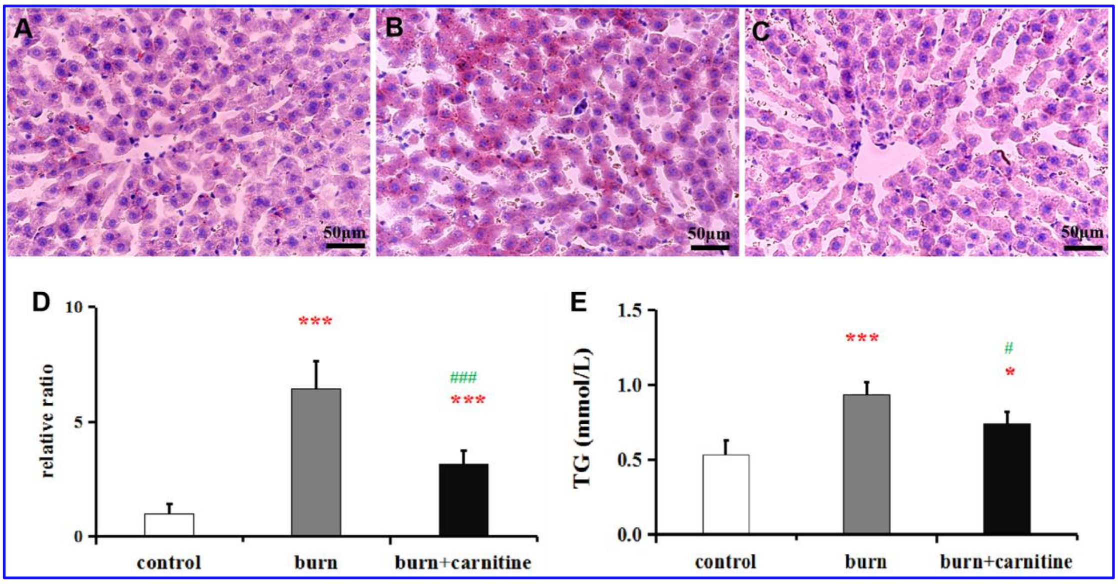

Fig. S3. Effects of exogenous carnitine on hepatic TG levels in burned rats

Representative Oil Red O staining images of the liver tissues from rats in the control (A), burn (B), and burn + carnitine (C) groups at 24 h post burn. Ten random fields were selected for each group, and the area of stained lipid droplets was calculated. The relative Oil Red O staining area in the liver tissue is shown (D). TG levels in the liver tissues are presented in (E). Data are expressed as means ± SD; n = 6 rats. *, $P < 0.05$ and ***, $P < 0.001$ vs. the control group; #, $P < 0.05$; ##, $P < 0.01$; and ###, $P < 0.001$ vs. the burn group.

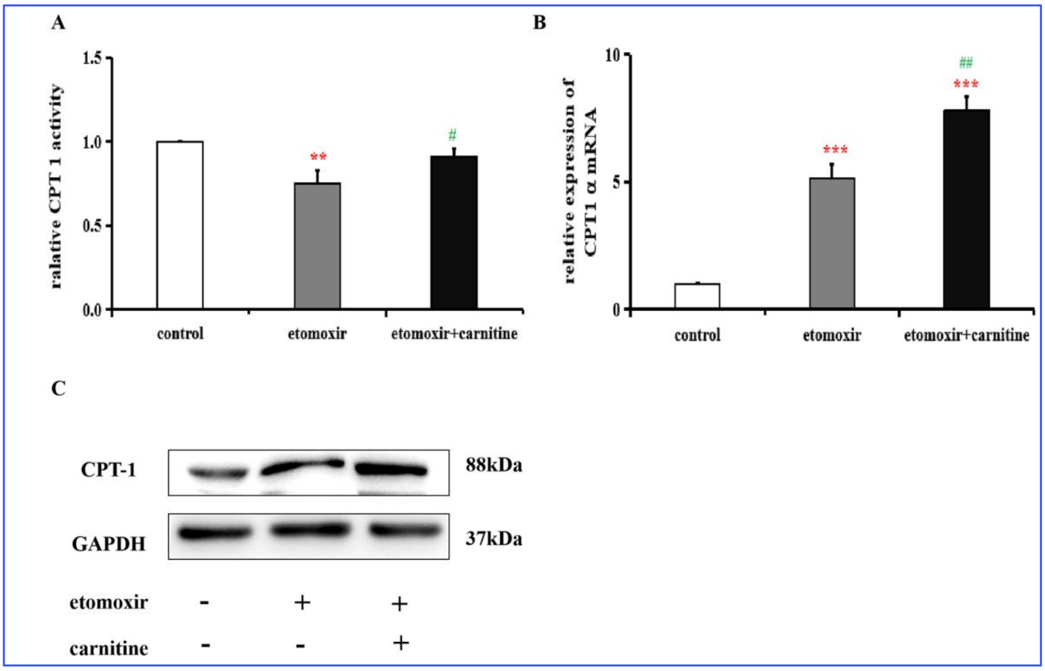

Fig. S4. Effects of carnitine on CPT1 activity and CPT1 expression in vitro

HepG2 cells were treated with or without 0.1 mM etomoxir or with 0.1 mM etomoxir + 0.2 mM L-carnitine for 24 h. (A) CPT1 activity and the mRNA (B) and protein (C) expression of CPT1α was detected via RT-qPCR and western blotting. Data are expressed as means ± SD; n = 3. **, $P < 0.01$ and ***, $P < 0.001$ vs. the control group; #, $P < 0.05$; ##, $P < 0.01$; and ###, $P < 0.001$ vs. the etomoxir group.

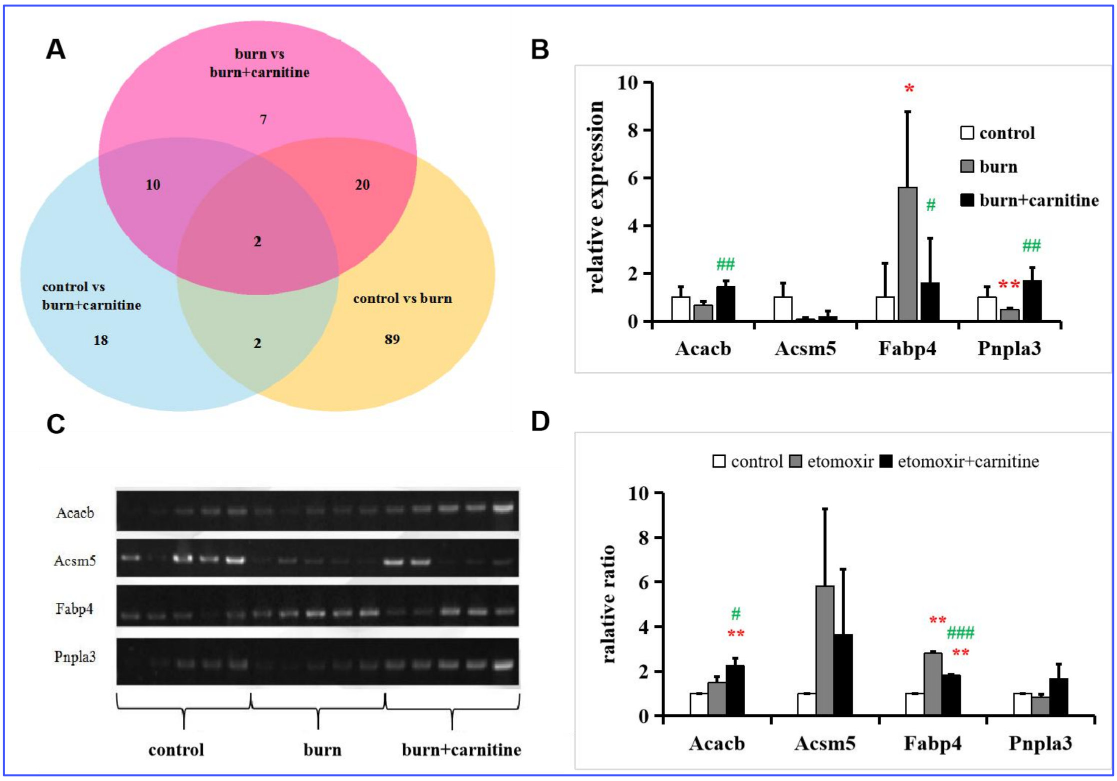

Fig. S5. Gene expression analysis in burned rats using high-throughput sequencing

(A) The yellow and red crosses are the overlapping differentially expressed genes in the Venn diagram. (B) The expression levels of Acacb, Acsm5, Fabp4, and Pnpla3 in the livers of burned rats were measured via RT-qPCR. (C) Agarose gel electrophoresis. Data are shown as means ± SD; n = 6. *, $P < 0.05$ and ***, $P < 0.001$, vs. the control group; #, $P < 0.05$ and ##, $P < 0.01$ vs. the burn group. (D) HepG2 cells were treated with 0.1 mM etomoxir or with 0.1 mM etomoxir + 0.2 mM carnitine for 24 h, and then the expression levels of Acacb, Acsm5, Fabp4, and Pnpla3 were assessed via RT-qPCR. Data are expressed as means ± SD; n = 3. *, $P < 0.05$ and **, $P < 0.01$ vs. the control group; #, $P < 0.05$ and ###, $P < 0.001$ vs. the etomoxir group.
